# Nurse-Led Randomized Controlled Trials in the Perioperative Setting: A Scoping Review

**DOI:** 10.2147/JMDH.S255785

**Published:** 2020-07-21

**Authors:** Judy Munday, Niall Higgins, Saira Mathew, Lizanne Dalgleish, Anthony S Batterbury, Luke Burgess, Jill Campbell, Lori J Delaney, Bronwyn R Griffin, James A Hughes, Jessica Ingleman, Samantha Keogh, Fiona Coyer

**Affiliations:** 1 Centre for Healthcare Transformation, Faculty of Health, Queensland University of Technology (QUT), Brisbane, QLD, Australia; 2 School of Nursing, Queensland University of Technology (QUT), Brisbane, QLD, Australia; 3 Department of Health and Nursing Science, University of Agder, Grimstad, 4879, Norway; 4 Mater Research Institute-UQ, South Brisbane, QLD 4101, Australia; 5 Royal Brisbane and Women’s Hospital, Herston, QLD 4029, Australia; 6 Colleges of Health and Medicine, Australian National University, Acton, ACT 2601, Australia; 7 Alliance for Vascular Access Teaching and Research, Griffith University, Brisbane, QLD, 4111, Australia

**Keywords:** perioperative, nursing, randomized controlled trial, scoping review

## Abstract

**Purpose:**

Nurses provide care at each phase of the complex, perioperative pathway and are well placed to identify areas of care requiring investigation in randomized controlled trials. Yet, currently, the scope of nurse-led randomized controlled trials conducted within the perioperative setting are unknown. This scoping review aims to identify areas of perioperative care in which nurse-led randomized controlled trials have been conducted, to identify issues impacting upon the quality of these trials and identify gaps for future investigation.

**Methods:**

This scoping review was conducted in reference to the Preferred Reporting Items for Systematic Reviews and Meta-Analyses Extension for Scoping Reviews. Searches were conducted in PubMed, Embase, Cumulative Index for Nursing and Allied Health Literature and the Cochrane Central Register of Controlled Trials, with a date range of 2014–19. Sources of unpublished literature included Open Grey, and ProQuest Dissertation and Theses, Clinical Trials.gov and the Australian and New Zealand Clinical Trials Registry. After title and abstract checking, full-text retrieval and data extraction, studies were appraised using the Joanna Briggs Institute Critical Appraisal Checklists for randomized controlled trials. Data were synthesized according to the main objectives. Key information was tabulated.

**Results:**

From the 86 included studies, key areas where nurses have led randomized controlled trials include patient or caregiver anxiety; postoperative pain relief; surgical site infection prevention: patient and caregiver knowledge; perioperative hypothermia prevention; postoperative nausea and vomiting; in addition to other diverse outcomes. Issues impacting upon quality (including poorly reported randomization), and gaps for future investigation (including a focus on vulnerable populations), are evident.

**Conclusion:**

Nurse-led randomized controlled trials in the perioperative setting have focused on key areas of perioperative care. Yet, opportunities exist for nurses to lead experimental research in other perioperative priority areas and within different populations that have been neglected, such as in the population of older adults undergoing surgery.

## Introduction

Health care providers are facing pressure to provide effective services to an increasing population with often limited resources.^1^ This pressure to provide more with less is evident within the provision of perioperative care. As morbidity increases, so does the complexity of surgery and the pressure upon resources in this highly technical, resource-intensive, fast-paced acute clinical environment.

For most patients, the experience of undergoing a surgical procedure represents a significant life event. During this critical period, health care practitioners are entrusted to advocate for and maintain the safety of patients when they are removed from family and loved ones and unable to speak up for themselves due to anesthesia.^2^ A safe passage through surgery is the highest priority. However, it has been argued that – despite the amount of effort spent on developing interventions and policy in recent years – progress in optimising patient safety in perioperative care has been much slower than anticipated.^3^

Internationally, perioperative care is described in four distinct phases: pre-admission, the immediate preoperative (pre-anesthetic) phase, the intraoperative phase (during induction of anesthesia and surgery itself), and the immediate postoperative phase of care (prior to patients returning to ward areas).^4^ This multi-staged pathway necessarily involves care delivered by a range of health care professions: registered and enrolled nurses, surgeons, anesthetists, technicians, orderlies, and radiographers. However, nurses are a consistent presence at all phases of perioperative care and may work in multiple roles, including preoperative care, anesthetic assistance, intraoperative (scrub/scout), and immediate postoperative care roles. In some countries, other professions such as registered operating department practitioners (ODPs) take on perioperative roles.^5^ However, globally nurses have a ubiquitous presence in health care teams that provide perioperative care and are uniquely placed to understand critical points of care and patient concerns across the whole perioperative pathway. It is imperative that nurses ensure they are both driving health care improvements and identifying research priorities in this specialized field.

Experimental research underpins the assessment of the effectiveness of interventions, yet it is widely acknowledged that randomized controlled trials (the gold standard of experimental research) are expensive, resource-intensive and time-consuming.^6^ It is essential that time and finite resources are well spent on interventions that are effective, safe and acceptable to patients. Resources and funding to conduct research are difficult to obtain, and therefore it is imperative that resources are directed to areas where gaps in experimental research exist. Furthermore, there is a need to ensure that resources are directed toward research that will be conducted in a rigorous manner in order to ensure high quality and reliable findings.

### Experimental Research in the Perioperative Setting

The conduct of rigorous, randomized controlled trials is often inhibited by well-known factors such as cost, time and resources. There are also other challenges in conducting research within this complex, multidisciplinary field that are not widely acknowledged. For instance, many recent systematic reviews and meta-analyses of perioperative care lack sufficient detailed report of individual elements of care which may impact on or confound outcomes.^7^ Perioperative outcomes are influenced by a wide range of factors throughout the preoperative journey and need to account for the truly multidisciplinary nature of perioperative care, by including nursing as well as medical interventions during each phase of care in study designs.^6^,^8^ Therefore, the complexity of the perioperative pathway needs to be considered in both the design of primary studies and the assessment of these studies via systematic review. Authors have recently questioned the status of randomized controlled trials in remaining the “gold standard” design to inform perioperative decision-making.^8^,^9^ Several authors have suggested that carefully designed before-and-after (observational) studies can be used to inform perioperative decision-making, with the benefit of being less resource-intensive, and more indicative of the feasibility of implementing interventions in actual practice.^8^,^9^ However, well-conducted, randomized controlled trials offer the highest level of scrutiny, with the lowest level of bias and therefore the greatest benefits to our patients, and remain the gold standard of experimental studies.^6^

### Nurse-Led Research in the Perioperative Setting

The multidisciplinary nature of perioperative care can result in challenges for nurses when trying to implement evidence-based practice change, such as negotiating staff buy-in across large multidisciplinary groups.^10^,^11^ Challenges also exist for perioperative nurses engaging in primary research that is pertinent to the discipline, such as funding. Potential sources of funding for specifically nurse-led research may also be even more scarce given the seemingly limited lack of financial backing for perioperative research both locally and internationally.^12^ Yet, the importance of supporting perioperative nurses to undertake research is vital in both facilitating evidence-based change in this domain of care. Nurses must drive research priorities that are relevant to perioperative nursing care.^13^ Although perioperative nurse-led research may be increasing, the extent to which of these are nurse-led perioperative randomized controlled trials has not been evaluated.

## Methods

### Aim

The purpose of this scoping review is to identify in which domains of perioperative care nurses are leading experimental research.

### Objectives

The main objectives of the scoping review were the following:

- To identify in which domains of perioperative care nurse-led randomized controlled trials have been conducted.
- To analyse the issues impacting upon the quality of experimental research undertaken in the perioperative setting.
- To identify what, if any, gaps exist in nurse-led experimental research in the perioperative setting, thus identifying priorities for future research.

### Design

This scoping review was conducted in reference to the methodology set out by the Joanna Briggs Institute,^14^ with the framework developed by Arksey and O’Malley^15^ and reported according to the Preferred Reporting Items for Systematic Reviews and Meta-Analyses Extension for Scoping Reviews (PRISMA-ScR).^16^ The scoping review methodology is appropriate for this question as it facilitates a broad exploration of perioperative care domains in which nurses are researching. This approach has been used successfully in similar reviews that have explored the scope of research undertaken in other specialised areas of health care.^17^,^20^ Scoping reviews are not eligible for registration with PROSPERO.

### Search Methods

A comprehensive search strategy was undertaken to find both published and unpublished (gray) literature in English from 2014 – May 2019, as per the recommendations for scoping reviews established by Peters et al.^14^ Only studies published in English were included due to lack of resources for translation. Databases for published literature included PubMed, Embase, Cumulative Index for Nursing and Allied Health Literature (CINAHL) and the Cochrane Central Register of Controlled Trials (CENTRAL). The search for unpublished literature utilised OpenGrey, and ProQuest Dissertation and Theses (PQDT). Searches for trials in progress were conducted using Clinical Trials.gov and the Australian and New Zealand Clinical Trials Registry (ANZCTR). Initial searches of PubMed and CINAHL were conducted to refine index terms and keywords, followed by a second search with keywords and index terms across all databases. Finally, perioperative nursing journals (Journal of PeriAnesthesia Nursing; Journal of Perioperative Practice: AORN Journal; Journal of Perioperative Nursing in Australia; Perioperative Care and Operating Room Management) were screened for additional randomized controlled trials across the date range.

Initial search terms for CINAHL were as follows:

1. “perioperative”
2. MH “Perioperative Care+”
3. MH “Perioperative Nursing+”
4. MH “Perioperative Period+”
5. MH “Preoperative Care+”
6. MH “Preoperative Period+”
7. MH “Intraoperative care+”
8. MH “Intraoperative Period+”
9. MH “Postoperative Care+”
10. MH “Postoperative Period+”
11. MH “Post Anesthesia Care+”
12. MH “Post Anesthesia Care Units+”
13. MH “Anesthetics+”
14. #1 OR #2 OR #3 OR #4 OR #5 OR #6 OR #7 OR #8 OR #9 OR #10 OR #11
15. MH “Randomized controlled trials+”
16. #12 AND #13

### Inclusion and Exclusion Criteria

Studies that met the following inclusion criteria were eligible for review:

Population: participants receiving care during one or more phases of the perioperative pathway: preoperatively, intraoperatively or immediately postoperatively.

Concept (study designs): only nurse-led randomized controlled study designs were included. To enable the identification of these particular trials, in-depth investigation of author names and qualifications were performed for those studies in which details were not listed on the abstract or full text. Other trials were included if known to be led by nursing academics but whose qualifications are not explicitly stated in the citation.

Context: studies focused on perioperative care including the preoperative, intraoperative or immediate postoperative setting.

### Screening and Eligibility Process

Four reviewers conducted screening of titles and abstracts to identify relevant papers for full-text retrieval (JM, NH, LD, SM). Full texts were then screened for eligibility against the inclusion criteria by the authorship team using a verification form developed for this purpose (Supplementary File 1).

### Data Charting Process

**Figure 1:**
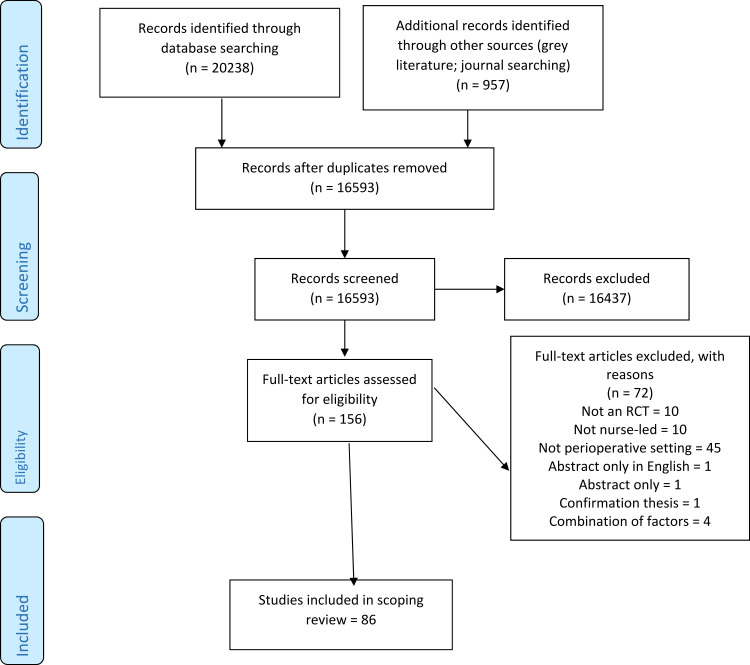
PRISMA flow diagram.

A flow chart was generated to indicate the papers included in the review at each stage, as per the PRISMA guidelines (Figure 1).^16^ A data charting form was developed to record and extract study characteristics and variables relevant to the review question (Supplementary File 2). Pairs of reviewers undertook data extraction independently for each article and mediated by a third where there was a lack of agreement.

### Critical Appraisal

Studies identified as relevant to the review were assessed for quality using the JBI Critical Appraisal Checklists for Randomized Controlled Trials.^21^ Whilst quality assessment is not considered mandatory in scoping reviews, undertaking this process assisted in identifying common issues that influenced or undermined the quality of randomized controlled trials in the perioperative setting. Pairs of reviewers also assessed each included study for quality, with disagreements resolved through discussion and consensus. Where agreement was not resolved through this process, an independent third reviewer was utilized.

### Synthesis

Following data extraction and quality assessment, key information from each study was tabulated to assist in determining country of origin, interventions, primary outcomes, surgical population, sample size and funding source (Supplementary File 3). Studies were organised according to the primary outcome in order to identify domains of perioperative care. Within each primary outcome, the interventions of interest and the study population assisted in determining gaps in phases of care or where study populations had not been included.

To analyse factors influencing the overall quality of included studies, common quality indicators were synthesized according to the quality assessment checklist where studies had scored poorly.^21^ Areas of perioperative care where experimental nurse-led research is appropriate but not yet evident were identified. Data synthesis and analysis were discussed within the authorship team to ensure consensus and that all relevant themes within the review questions were identified. Results are presented in table form, to provide an overview of all included studies as per the data extraction (charting) form.

## Results

Eighty-six studies were included in the final review (Figure 1). The included studies were geographically widespread (Table 1). The region of origin with the most included RCTs was North America (n = 28)^22^,^49^ followed by Europe (n=26),^50^,^75^ Asia (n=15),^76^,^90^ the Middle East (n=7),^91^,^97^ Oceania^98^,^102^ and South America (both n=5).^103^,^107^

**Table 1:** Randomized Controlled Trials by Country and Region

Region Country	Number (n, % of Total)
**Oceania** Australia	5 (5.8)
**South America** Brazil	5 (5.8)
**North America**	
Canada	3
USA	25
Total	28 (33)
**Asia**	
China	3
Hong Kong	1
India	1
Singapore	1
South Korea	3
Taiwan	6*
Total	15* (17)
**Europe**	
Croatia	1
Denmark	2
France	1
Greece	1
Italy	4
Norway	1
Spain	3
Sweden	4
Turkey	9
Total	26 (30)
**Middle East**	
Iran	6
United Arab Emirates (UAE)	1
Total	7 (8)
Overall Total	86

**Note:** *Duplication of one study into two publications noted in this group.

### Domains of Perioperative Care Addressed by Nurse-Led Randomized Controlled Trials

Six main domains of perioperative care, addressed by nurse-led RCTs were identified: (i) prevention of caregiver and patient anxiety; (ii) perioperative hypothermia prevention and temperature monitoring; (iii) postoperative pain relief; (iv) postoperative nausea and vomiting (PONV) prevention and treatment; (v) prevention of surgical site infection (SSI): (vi) patient and parental knowledge; in addition to other diverse clinical outcomes (Supplementary File 3).

#### Prevention of Caregiver and Patient Anxiety

Prevention of anxiety, both from the patient and caregivers’ perspective, was the most common primary outcome of interest, accounting for over a fifth of studies (n=20, 23%).^32^,^37^,^38^,^49^,^53^,^54^,^57^,^58^,^59^,^63^,^70^,^71^,^79^,^81^,^91^,^93^,^94^,^103^,^105^,^108^ Prevention of anxiety was a secondary outcome of interest in a further nine (10%) studies.^22^,^23^,^25^,^47^,^50^,^55^,^69^,^73^,^80^ Of the studies including anxiety prevention as the primary outcome, nine studies (47%) were focused on adult patients;^32^,^38^,^53^,^57^,^59^,^71^,^81^,^94^,^105^ nine were focused on pediatric patients,^37^,^49^,^54^,^63^,^79^,^91^,^93^,^103^,^108^ (with four of these also including caregivers as a sub-population,^37^,^49^,^54^,^108^ and another focused on adolescents^37^); one study concentrated solely on caregiver (parent) anxiety.^70^ The interventions of interest included music;^32^,^58^,^59^,^71^,^103^ education (including videos);^37^,^70^,^81^,^94^ visiting preoperative facilities;^54^ play;^79^,^91^,^93^,^108^ relaxation and sounds from nature;^57^ aromatherapy;^53^ photographic displays;^58^ distraction versus midazolam;^49^ therapeutic listening;^105^ different timings of communication^38^ and an application with Clown Doctors.^63^

#### Perioperative Hypothermia Prevention and Temperature Monitoring

Thirteen published studies (15% of included studies) had a primary outcome of preventing perioperative hypothermia or temperature monitoring.^35^,^46^,^56^,^74^,^82^,^85^,^86^,^87^,^96^,^98^,^99^,^100^,^104^ However, one study was published twice in two different journals.^85^,^87^ Active warming (comprising forced air, thermal gown, intravenous (IV) fluid warming or underbody warming) and passive warming strategies (reflective versus cotton blankets or cloths) were tested in various combinations. All perioperative hypothermia studies were conducted in the adult population, but within different surgical specialities: interventional cardiovascular procedures;^99^ gastro-intestinal or thoracic surgery;^85^,^87^ obstetrics;^35^,^98^ laparoscopic cholecystectomy;^96^ colorectal surgery;^56^ gynaecology;^104^ cardiovascular^74^ or multiple specialities.^82^,^100^ One study assessed skin temperatures after blankets warmed to different temperatures in a population of healthy volunteers.^46^

#### Postoperative Pain Relief

Postoperative pain relief was the third most common primary outcome of interest (n=13, 15% of included studies),^22^,^24^,^31^,^34^,^36^,^41^,^50^,^51^,^55^,^62^,^65^,^72^,^92^ and a secondary outcome in 13 studies (15%).^35^,^40^,^47^,^52^,^60^,^69^,^75^,^76^,^79^,^81^,^86^,^87^ Interventions of interest in the studies where pain was the primary outcome included hypnosis;^55^ anaesthetic technique (for hysteroscopy);^51^ play;^72^ Reiki;^34^ premedication and information;^50^ different routes of paracetamol administration;^41^,^62^ cold application;^65^ guided imagery and relaxation;^22^ positioning and early sandbag removal (post-coronary angiography);^92^ room air versus carbon dioxide (CO_2_) insufflation;^24^,^31^ and bed positioning.^36^ Nine studies had adult participants,^31^,^34^,^36^,^41^,^50^,^51^,^62^,^65^,^92^ two were pediatric based,^55^,^72^ and one study focused on adolescents.^22^

#### Postoperative Nausea and Vomiting (PONV) Prevention and Treatment

Eleven studies (13% of included studies) focused on the prevention or treatment of PONV. Six studies tested pericardium 6 (P6) acupressure;^29^,^43^,^64^,^69^,^73^,^89^ two studies tested aromatherapy with or without additional therapies;^39^,^48^ one study tested early hydration;^90^ one study tested an individualised preoperative education intervention^40^ and one study tested different doses of promethazine.^44^

#### Prevention of Surgical Site Infection (SSI)

Five studies (6% of included studies) focused on SSI prevention as the primary outcome, using a variety of interventions: postoperative shampooing;^66^ preoperative 2% chlorhexidine gluconate skin preparation cloths;^42^ silver impregnated versus standard dry sterile dressings (cardiac surgery):^26^ hair shaving techniques;^61^ different antiseptic methods.^88^

#### Patient and Parental Knowledge

The primary outcome of interest for five studies (6% of included studies) was patient or parental knowledge.^23^,^67^,^80^,^106^,^107^ Predominantly, these studies tested the effect of video or multimodal education interventions: video resources;^23^,^80^,^106^,^107^ multimethod education or information booklets versus questions.^67^ Three studies were interested in adult patient knowledge,^67^,^80^,^106^ two on parental knowledge.^23^,^107^

#### Other Clinical Outcomes

A wide variety of other clinical practices were investigated as primary outcomes in the identified RCTs (Supplementary file 3).^25^,^27^,^28^,^30^,^33^,^45^,^47^,^52^,^60^,^68^,^75^,^77^,^78^,^95^,^101^,^102^

### Perioperative Research Populations and Phases of Care Addressed by Nurse-Led Randomized Controlled Trial Designs

#### Study Populations

Predominantly, studies were focused on the adult population (n= 71, 83%), with 10 studies focusing on pediatrics as the population of interest (12%). Four studies included both caregivers and children as the population of interest,^23^,^47^,^49^,^54^ whilst one study focused on caregivers only.^107^ Two studies focused on adolescents,^22^,^37^ and one study included both adults and children.^84^ Although older adults (>75 years) were included in some studies^52^,^60^,^62^ they were not specifically identified as the target population in any of the included studies.

#### Phases of Care

Over half of studies involved interventions that were delivered during the preoperative phase of care (n=41, 48%); 13 studies delivered interventions during the intraoperative phase (n = 13, 15%);^24^,^26^,^31^,^43^,^46^,^51^,^74^,^75^,^86^,^92^,^97^,^99^,^101^ 13 studies (15%) delivered interventions solely in the postoperative phase,^36^,^39^,^44^,^47^,^48^,^60^,^66^,^68^,^73^,^77^,^82^,^90^,^107^ Supplementary file 3; eight studies (9%) were based on interventions that were delivered during multiple phases of the perioperative pathway.^34^,^35^,^42^,^56^,^61^,^76^,^85^,^96^ Almost half of the included studies assessed outcomes at multiple phases of the perioperative pathway (n = 34, 40%), whilst 24 studies (28%) assessed postoperative outcomes extending beyond the immediate PACU phase.^26^,^27^,^34^,^35^,^39^,^40^,^41^,^43^,^45^,^48^,^51^,^55^,^61^,^62^,^64^,^66^,^69^,^73^,^89^,^90^,^92^,^99^,^102^,^109^ Five studies (6%) assessed outcomes only during the preoperative phase,^57^,^58^,^71^,^103^,^106^ whilst only four studies assessed outcomes at a single phase of intraoperative care (n=4, 6%),^33^,^46^,^56^,^59^,^74^ and seven studies assessed outcomes during PACU care only (n=7, 8%).^24^,^44^,^47^,^68^,^82^,^100^,^109^

### Issues Impacting Upon the Quality of Experimental Research Undertaken in the Perioperative Setting

Issues impacting upon the quality of RCTs included in this review were related predominantly to the reporting of blinding techniques. Blinding of participants was unclear or not implemented in 79% of included studies (n=68); blinding of those delivering the intervention was not utilised or was unclear in 80% (n=69) studies, and blinding of outcome assessors was not utilized or was unclear in 73% (n=63) of included studies. Many studies did acknowledge the reasons for lack of blinding and most often this was related to the nature of the intervention under study: yet, most often lack of blinding of one or more key groups was not discussed or acknowledged as a limitation.

In addition, a lack of, or unclear randomization was found in just over a quarter of included studies (35%, n=31). Similarly, a high number of included studies were assessed as having incomplete follow-up or there was inadequate analysis or description of differences between groups (32%, n =28). Duplication of study results was also found in one instance, where the same study was published in different journals with a different author order.^85^,^87^

## Discussion

To our knowledge, this is the first scoping review to investigate the range of nurse-led randomized controlled trials conducted in the perioperative setting. Geographically, this review has revealed that North America contributed the highest number of studies to this review, with the United States the most prolific individual country in terms of conducting nurse-led perioperative RCTs in the last 5 years. This contrasts with a recent scoping review of RCTs and quasi-experimental studies published in nursing journals, whereby Taiwanese nursing researchers were found to have published the most frequently in nursing journals.^110^ However, our review also included studies that, although nurse-led, were published in journals that were not specifically nursing-focused, and only focused on RCTs which was appropriate to address the review question. Similarly, though, our review also found no African studies for inclusion.^110^ This may be unsurprising given that a 2015 scoping review of clinical nursing and midwifery research in African countries found that, at the time of the review, most included research was qualitative, and focused on primary or secondary prevention of cancer.^111^ Additional obstacles to conduct and publication of nursing research in this region include a lack of resources (including funding, library access, equipment and collaborators) and political and civil unrest.^112^

This review of 86 studies revealed that there are six clearly identifiable areas in which nurses are leading experimental research (specifically RCTs) relevant to perioperative care. The most common primary outcome across included studies was the prevention of anxiety and this was investigated using a range of supportive interventions. Given how commonly preoperative anxiety is experienced, and the detrimental patient outcomes associated with anxiety,^54^,^93^ this may be justified despite anxiety prevention not being a stated priority by professional associations. The investigation of supportive or complementary therapies may be reflective of the growing interest in complementary therapies in health care more broadly.

The quality issues noted in this review, in which a large proportion of studies assessed the effectiveness of supportive therapies, indicate that nursing researchers are utilising facets of the randomized controlled study design adaptively (and creatively). Given the expense and resources required to conduct RCTs, it is imperative for nurses to ensure that these resources are well spent on trials that are well conducted and provide useful findings. At this stage, it may be pertinent for the focus on anxiety prevention to shift from primary research to translation into practice.

Almost half of the included studies (47%) assessed interventions that were delivered during the preoperative phase. A moderate number (n=13, 15%) delivered interventions during the intraoperative phase, but due to the nature of the interventions and outcomes under study – for example, the focus on anxiety reduction which would be difficult to assess intraoperatively due to anesthesia – few studies assessed outcomes during the intraoperative phase of care (n=4, 5%). This gap in the literature is an opportunity for nurses to design experimental studies that measure the outcomes of interventions and outcomes related to intraoperative or procedural nursing care. Despite anxiety prevention being the most common outcome in the included studies, one did highlight that further investigation with teens or adolescents is worthy of future study.^54^

Whilst some regions and countries have established perioperative research priorities,^113^,^115^ an international consensus is not evident. The lack of consensus may be influenced by the diverse and differing needs between developed and under-developed regions, but also reflects the variation in the processes used to determine the published perioperative priorities (including the variation in stakeholder involvement). The perioperative pathway is complex, multi-staged and involves numerous health professions in the delivery of care. Therefore, it is logical that any work to establish areas of perioperative care that requires a stronger evidence base needs to ensure multidisciplinary input – as well as ensuring that health care consumers also have input.

In the United Kingdom (UK), the National Institute of Academic Anaesthesia and James Lind Alliance (JLA) Research Priority Setting Partnership’s agreed 10 anaesthetic and perioperative care priorities include a range of issues. These range from the study of the term effects of anesthesia, to establishing “success” measures for perioperative care.^113^ The authors determined that specific care and physiological questions were ranked more highly by clinicians, whereas lay stakeholders ranked communication and long-term outcomes of anesthesia more highly.^113^ Similarly, Biccard et al’s Delphi study of perioperative investigators in South Africa, whilst recognising the need for a co-ordinated perioperative research agenda, established national priorities that focused on a wide range of quite specific clinical care aspects although lay input into this process was not evident.^115^ The failure to investigate outcomes that matter to patients within pragmatic trials is not unique to perioperative care.^6^ Nonetheless, the primary outcomes of anxiety prevention and knowledge generation identified in this review align more closely with lay stakeholder-identified priorities related to communication,^26^ which may be unsurprising given that patient advocacy is a key nursing role.

This review also found that safety outcomes received minimal attention in the nurse-led trial research included in this review. It has also been argued that safety outcomes, having also been neglected, should also be reported in pragmatic trials in the perioperative setting.^6^ Within the perioperative nursing field, Steelman’s top 10 patient safety priority areas, established by perioperative nurses in the USA, identify only one of the primary outcomes of interest found in the included studies in this review as a safety concern (perioperative hypothermia prevention).^116^ However, many of these safety concerns may not lend themselves as a focus of experimental research due to being rare events (for example, wrong-site surgery; prevention of retained surgical items; surgical fires) whilst others are less so (medication errors; pressure injuries).^116^ A number of aspects of perioperative hypothermia prevention are also identified in the Association of periOperative Registered Nurses (AORN) 2019 Research Gaps.^117^ The AORN’s Research Priorities for 2018–2023 focus on patient education practices as well as the need to improve outcomes for vulnerable populations.^114^

The outcomes from this review of nurse-led RCTs do align, to some degree, with care priorities established by the Australian Government that are published in clinical indicators and guidelines. In the Australian setting, perioperative hypothermia (measured as the number of patients arriving into PACU with a temperature of less than 36°Celsius), pain, PONV, surgical site infection and post-dural puncture headache – all outcomes of interest in the included studies – are key clinical indicators assessed by the Australian Council on Healthcare Standards in the most recent Australasian Clinical Indicator Report for 2010–17.^118^ This report highlights that, for some areas, meeting the Key Performance Indicators has been problematic. For example, in 2017 there was an increased incidence of perioperative hypothermia reported.^118^ Therefore, it can be argued that the continued focus on developing strategies to manage this condition is warranted.

All health care professionals leading experimental perioperative research need to ensure that the populations upon which research is focused are reflective of the needs of the surgical populations. As mentioned, no studies specifically focused on the needs of older adults were found in this review. Studies of younger, fitter populations may not be truly reflective of surgical populations outside of trial settings; thus, the practical application of research findings is reduced, and the interests of the older adults receiving surgical care may not be met. This need has been evident over the last ten years. In 2010, a large multicentre, prospective observational study of older adults undergoing surgery in Australia and New Zealand highlighted that complications and mortality amongst this cohort were prevalent, and strategies were urgently needed to address these issues.^119^ However, nurse-led randomized controlled trials in the perioperative setting do not reflect the trend of focusing on older adults, and patients with cancer, which were reported more broadly in nurse-led experimental research across clinical settings.^110^

This review has also revealed that common quality indicators are problematic in the conduct of RCTs in this setting. Unclear randomization was evident across the majority of studies, despite the inclusion criteria only specifying randomized controlled designs. There was a lack of blinding in the included studies. In the studies where blinding was implemented, the method of blinding varied considerably. Successful blinding may have occurred for the participant, those delivering interventions and/or the outcome assessors. Whilst a number of studies acknowledged and provided an explanation for a lack of blinding, many other studies either reported but did not explain, or did not acknowledge the lack of blinding at all. Where acknowledged, most often blinding was not achieved due to the nature of the intervention. This is perhaps unsurprising, given that most of the interventions were delivered and/or outcomes assessed, at time points of care where patients were awake. It is acknowledged that interventions such as the use of forced air warming, or some complementary therapies, are extremely problematic when trying to include effective blinding techniques for participants.^99^ Nonetheless, bias related to lack of participant blinding may be offset by the assessment of objective outcome measures, and the use of outcome assessor blinding where possible.^120^

### Limitations

There is potential that some nurse-led RCTs meeting the inclusion criteria have been inadvertently missed, despite our extensive and thorough search process. The process of identifying nurse-led studies was complex during the search phase of this review. Not all studies clearly identified the professional background of authors. This meant that additional searches of the primary author’s name were, in some instances, needed to identify whether or not studies were nurse-led.

This review also only provides a picture of randomized controlled studies conducted by nurses in the last 5 years. Quasi-experimental, observational study designs, qualitative studies were not included, nor were secondary analyses such as systematic reviews and meta-analyses. Therefore, this review cannot provide an indication of the non-experimental or synthesised body of evidence generated by nurses in this clinical setting. We also only included studies published in English. Future studies may seek to investigate the body of nurse-led research conducted using these study designs to gain a more inclusive snapshot of research in this clinical setting.

## Conclusions

This scoping review has identified clear areas of perioperative care that have been the focus of nurse-led randomized controlled trials. The emphasis has been on supportive care of both patients, and caregivers. Most conducted research has involved multiple phases of care, across the perioperative pathway. Significant issues affecting the quality of experimental nurse-led research conducted in the perioperative setting have also been identified, mainly relating to blinding and randomisation. Acknowledging these issues provides opportunities for maximising research quality in nurse-led experimental research. Gaps in perioperative nursing research exist in focused assessment of intraoperative or procedural aspects of care, patient safety outcomes and care of vulnerable groups. Opportunities also exist for nurses to contribute to multidisciplinary research priority setting in the perioperative field and focus on the translation of evidence to practice in areas such as anxiety prevention where further extensive experimental research may not be warranted. Priority settings must also include patients and caregivers as stakeholders to ensure that we are meeting their needs.

## Abbreviations

APAIS, Amsterdam Preoperative Anxiety and Information Scale; AORN, Association of periOperative Registered Nurses; ASA, American Society of Anesthesiologists; ASPAN, American Society of PeriAnesthesia Nurses; BPU, bordered polyurethrane; CINAHL, Cumulative Index for Nursing and Allied Health Literature; CO_2,_ carbon dioxide; CRBSI, catheter-related bloodstream infection; DBP, diastolic blood pressure; ENT, ear, nose and throat; FLACC, Faces, Legs, Activity, Cry, Consolability Scale; GSQS, Groningen’s Sleep Quality Scale; HR, heart rate; JBI, Joanna Briggs Institute; IV, intravenous; LOS, length of stay; MAP, mean arterial pressure; mYPas, modified Yale Preoperative Anxiety Scale; mYPAS-SF, modified Yale Preoperative Anxiety Scale Short Form; NHMRC, National Health and Medical Research Council; NRS, Numerical Visual Anxiety Scale; NVAAS, Numerical Visual Analog Anxiety Scale; OT, operating theatre; P6, pericardium 6; PACU, post anesthetic care unit; PCA, patient-controlled analgesia; PONV, post-operative nausea and vomiting; RCT, randomized controlled trial; RR, respiratory rate; SBP, systolic blood pressure; SSD, sutureless securement device; STAI, State-Trait Anxiety Inventory; STAIC, State-Trait Anxiety Inventory for Children; STAI-Y, State-Trait Anxiety Inventory (Form Y); STTI, Sigma Theta Tau International; TA, tissue adhesive; VAS, Visual Analog Scale; Y-PAS, Yale Preoperative Anxiety Scale.
